# Plant Cell and Organism Development 2.0

**DOI:** 10.3390/ijms23031885

**Published:** 2022-02-08

**Authors:** Robert Hasterok, Alexander Betekhtin

**Affiliations:** Plant Cytogenetics and Molecular Biology Group, Institute of Biology, Biotechnology and Environmental Protection, Faculty of Natural Sciences, University of Silesia in Katowice, 40-032 Katowice, Poland

In the editorial summarising the first edition of the Special Issue on “Plant Cell and Organism Development”, we listed the key features that make plants a unique and fascinating group of living organisms [1]. However, current climate changes are affecting plants in their natural environments and those that are under cultivation. Global warming will likely redefine many plant habitats regarding temperature, water availability, soil erosion, salinity and other abiotic and biotic parameters. The ever-growing human population and increasing food demand require more extensive or more efficient agriculture, all within the limited capacity of our planet. For those and many other reasons, comprehensive research on plants has become more vital than ever.

Considering the fundamental importance of plant research and encouraged by the significant interest of the research community in the first edition, we decided to continue with “Plant Cell and Organism Development 2.0”. The second edition consists of 25 peer-reviewed papers, 20 articles and 5 reviews that provide a comprehensive state-of-the-art view into various aspects of experimental plant biology in vivo and in vitro. Compared with the first edition, we can see the increasing interest of the contributors not only in model plants but also in cultivated ones. The authors focus on many agronomically important traits of different plant species.

In the first article, Yu et al. [2] analyse 42 *Membrane Attack Complex and Perforin* (*MACPF*) genes from 6 species that represent the major Poaceae clades for which complete whole-genome information is available. Their phylogenetic, domain organisation, motif composition, gene structure and synteny analyses revealed the presence of four highly conserved groups and an uneven distribution of the *MACPF* genes along the grass chromosomes. Based on the results of intergenomic collinearity analyses, the authors postulated the importance of segmental and dispersed duplication events for the expansion and functional diversification of this gene family in Poaceae. Using transcriptomic approaches, they also revealed a higher expression of the *MACPF* genes in the vegetative tissues and their upregulation in response to various abiotic and biotic stresses. This study provides a foundation for the further functional characterisation of Poaceae *MACPF* genes during development and adaptation to stress.

The article by Nowak et al. [3] highlights the importance of the interplay between transcription factors (TF) and microRNAs as a prerequisite for better understanding how the cell transcriptome is fine-tuned regarding somatic embryogenesis (SE) control. Using an embryogenic culture of *Arabidopsis thaliana* (Arabidopsis), the authors analysed the regulatory relationships between AGL15 TF and miR156, both of which are known to be involved in SE induction. They revealed that AGL15 not only contributes to the activation of *MIR156* gene transcription but might also restrict the abundance of mature miR156 by repressing the genes that are responsible for miRNA biogenesis. The use of a well-known histone deacetylase inhibitor, trichostatin A, indicated that the latter effect could be assured via the co-operative action of the histone deacetylases HDAC6 and HDAC9 with AGL15. The authors conclude that similar to the findings in mouse and human cells, histone acetylation seems to control miRNA during the reprogramming of somatic plant cells towards pluripotency and that AGL15 might play an essential regulatory role in this process.

In the next article, Grzybkowska et al. [4] focus on the auxin-induced reprogramming of plant cells that is linked to the epigenetic modifications during SE. They revealed that treating Arabidopsis explants with auxin affected the expression and methylation patterns of *LEC1*, *LEC2*, *WUS* and *AGL15*, all of which are *TF* genes that are known to be critical SE induction regulators. While stable hypermethylation was observed in the promoter regions and gene bodies of the embryogenic culture, the promoters were characterised by a significantly higher methylation. The general conclusion is that elucidating how DNA methylation regulates genes during the embryogenic transition of somatic cells requires a further investigation of the complex interplay among the diverse epigenetic processes that control the SE-regulatory genes in response to auxin.

As per their article, Juzon et al. [5] provide a functional analysis of the photosynthetic apparatus in the drought-stressed oat × maize chromosomal addition (OMA) lines, which are hybrids between C3 and C4 plants. After 14 days of drought, they found that most of the OMA lines exhibited a better photosynthetic efficiency than the oat plants. The authors concluded that it was rather the retention of a specific maize chromosome and its interaction with the oat genome that was responsible for the expected changes in the hybrid functioning rather than the number of retained chromosomes. This study sheds new light on the complexity of interspecific hybrid functioning and some of the consequences of preserving alien chromosomes and also raises questions as to what extent these and similar hybrids can contribute to crop improvement.

In their comprehensive review, Tetlow and Bertoft [6] discuss the current knowledge about starch, which is the primary energy store in most plants and one of the primary carbohydrates in human nutrition. They focused on various aspects of the starch granule architecture and the starch biosynthesis pathway regarding the building block-backbone model of the starch structure. One of the key conclusions is that although there is plenty of information about the initial linear structures that are produced during the initiation of starch granules, little is known about the subsequent stages of granule formation. While enzymatic reactions and the smaller-scale structures such as building blocks that they produce are well identified, understanding the biological factors and self-assembly mechanisms that are responsible for higher-order structures such as blocklets and growth rings remains elusive.

Kuczak and Kurczyńska [7] investigate whether there is a relationship between the presence or absence of selected cell wall components and changes in the cell fate during SE induction in *Daucus carota* (carrot). Using the immunolocalisation of antibodies that target selected pectin, arabinogalactan protein (AGP) and extensin (EXT) epitopes, they observed that some pectin and AGP epitopes could be considered to be positive or negative markers of SE. In contrast, EXTs do not seem to contribute to the cell reprogramming in this species. These findings provide a comprehensive spatio-temporal description of changes in the wall composition in cells that are changing their fate.

The article by Njaci et al. [8] concerns a high-throughput transcriptome profiling approach for studying the defence mechanisms in a susceptible genotype of the important legume crop *Cajanus cajan* (pigeonpea) against *Helicoverpa armigera* (pod borer) and in its wild relative (*C. scarabaeoides*), which is resistant to this insect pest. The authors identified the key genes, signalling pathways and metabolites that are utilised by *C. scarabaeoides* in its defence against a pod borer infestation. These findings enhance the understanding of the response to insect damage in the *H. armigera*-resistant pigeonpea wild-relative at the molecular level and can be used in crop improvement by developing *C. cajan* cultivars that are resistant to this pest. They demonstrate the importance of wild crop relatives as crucial genetic resources and reservoirs in general terms.

Another article shows that Wolny et al. [9] analyse the impact of excessive NaCl salinity on the expression of the key cell wall genes, the cell cycle and histone modifications in *Brachypodium distachyon*. Using various morphological, cytological, histological and molecular approaches, they determined the NaCl threshold concentrations for seed germination and root elongation. The authors suggest that the increased AGP expression in response to salt could be linked with altered growth patterns, whereas the inhibition of the cell cycle activity in the plants that had been subjected to higher NaCl concentrations could indicate a toxic effect of the sodium ion accumulation. This study extends our knowledge of plant responses to salinity stress in an important model organism that is closely related to key temperate cereals and other economically important grass species. It can also be helpful for plant breeding programmes whose goal is to develop salt-tolerant crop cultivars.

The review by Mathe et al. [10] presents state-of-the-art information about the role of protein phosphatase PP2A in plants. This non-metal-dependent serine-threonine protein phosphatase is universal and is one of the most abundant protein phosphatases in eukaryotes, and it is involved in regulating many important cellular events. After a general introduction, they reviewed how PP2A regulates the microtubule-mediated vesicle traffic during cell plate formation in the context of plant cytokinesis. In the following section, the authors described the autophagy pathways that are related to PP2A and pay particular attention to the PP2A-mediated inhibition of the target of the rapamycin complex (TORC)-dependent signalling pathways. Then, they focused on the subcellular fate of the PIN proteins in relation to PP2A. In their concluding remarks, the authors asked some intriguing questions about future research directions that are linked with PP2A-regulated vesicle traffic and its developmental consequences.

In another review, Malerba and Cerana [11] summarise how cell cultures can help to clarify programmed cell death (PCD) in plants. This genetically controlled process is present in all multicellular organisms and is indispensable for eliminating unnecessary or detrimental cells for proper organism development. After introducing the topic of their review, the authors focused on the main advantages of studying PCD in plant cell cultures. In what followed, they discussed the PCD that is induced in cell cultures by biotic and abiotic stresses, respectively. In the concluding section, the authors hypothesise that apoptotic-like PCD and necrosis both operate in cultured plant cells with the former probably being the most common way in which cells die.

Based on the fact that in *Lilium lancifolium,* cytokinins can promote the initiation of an axillary meristem (AM) during the formation of bulbils, in their article, He et al. [12] elucidate the function of the cytokinin type-B response regulators (RRs). Because of its triploid nature, this ornamental and medicinal lily is primarily propagated asexually via bulbils that originate from AMs. The authors identified five type-B *LlRRs*, which encode the proteins with different amino acid numbers and revealed that these proteins localise in the nucleus and are widely expressed in various tissues. Using a functional analysis, they also found that all of them are involved in bulbil formation. Thus, this study contributes to a better understanding of the molecular mechanisms by which cytokinins regulate bulbil formation in lilies.

Hesami et al. [13] provide an extensive review of the current state of the knowledge and prospects of various aspects of tissue culture and genetic engineering in *Cannabis sativa* (cannabis), which is a plant with many applications in medicine, industry, recreation and agriculture. They review various in vitro culture-related methods such as a callus culture, cell suspension culture, hairy root culture and micropropagation, as well as strategies for improving in vitro cannabis culture procedures. Then, they focus on ploidy engineering in cannabis, followed by various genetic engineering approaches, both the ‘classical’ and the more recent ones such as clustered regularly interspaced short palindromic repeats-associated protein 9 (CRISPR/Cas9)-based genome editing. The authors conclude that modern biotechnologies will play an essential role in utilising the full potential of cannabis plants.

The article by Ngugi-Dawit et al. [14] is another study of an important legume, pigeonpea (*C. cajanus*), that examines the potential of it overcoming its susceptibility to the insect pest *H. armigera* by transferring the host plant resistance traits from its wild relatives. Using tandem mass spectrometry, the authors performed comparative quantitative analyses of the leaf proteomes from the ICPL 87 variety of *C. cajanus*, which is susceptible, and *C. scarabaeoides* IBS 3471, which is highly resistant to the pod borer. They revealed that the majority of the highly accumulated proteins in *C. scarabaeoides* were involved in processes such as ROS scavenging, signalling and the phenylpropanoid pathway. Thus, IBS 3471 seems to be a tempting candidate for improving cultivated pigeonpea.

In the following article, Zhu et al. [15] functionally characterise MLO4, one of mildew resistance locus O proteins in the roots of Arabidopsis. These proteins are found in many plant species and play an important role in various developmental processes. By analysing the interprotein interactions, the authors revealed that MLO4 interacts with CML12, which is a member of the calmodulin-like family. They also revealed that *mlo4* and *cml12* single mutants and *mlo4 cml12* double mutant had similar phenotypes in root growth. These results suggest the involvement of MLO4 in the root gravity response, possibly through an interaction with a CML protein.

The review by Roque-Borda [16] discusses the importance of plant germplasm cryostorage, which is a method for conserving biological material using ultra-low temperatures. They started by describing how plant genetic resources can be preserved in situ and ex situ. In what followed, the authors reviewed the cryoprotective agents, osmoprotective solutions, vitrification solutions and cryopreservation methods. The most extensive part of their review discussed the cryopreservation applications that are of agronomic interest and provided an overview of recently developed protocols. The final sections focused on various aspects of the stability of liquid-nitrogen-derived plant material. The authors conclude that including antioxidants and better characterising the specific genes that are involved in osmoprotection, membrane transport and dehydration tolerance should generate further advances in plant cryobiology.

As per their article, Oleszkiewicz et al. [17] elucidate the role of two *psy* paralogous genes in *D. carota*. These genes encode phytoene synthase, which is responsible for catalysing the initial steps in the main carotenoid biosynthesis pathway, and their number varies among plant species. Using CRISPR/Cas9 genome editing, the authors generated functional mutations in the *psy1* and *psy2* genes and demonstrated their impact on the accumulations of carotenoids and the mutual regulation of the expression of these genes. They also observed changes in the histology and ultrastructure of the carrot callus. One of the most original findings of this study is that impaired carotenoid biosynthesis is accompanied by cell wall remodelling.

In their article, Pinski et al. [18] provide a comprehensive quantitative and qualitative analysis of a cell wall proteome in the leaves of *B. distachyon* that had been subjected to high-temperature stress. They demonstrated that compared to the control (21 °C), the plants that had been grown for 24h at 40 °C had 46 cell wall proteins that were differentially abundant, of which four were over-accumulated while all of the others were under-accumulated. The authors also observed that the most significant changes concerned the proteins that act on the cell wall polysaccharides. These results suggest that a lower protease activity, lignification, expansion of the cell wall and changes in the architecture of its polymers and pectins, in particular, occur in response to high-temperature stress in the model grass.

Another article depicts how Hussain et al. [19] identify and functionally characterise two abscisic acid (ABA) response genes, *SMALLER TRICHOMES WITH VARIABLE BRANCHES* genes, *SVB* and *SVB2*, in Arabidopsis. They revealed that in the wild-type plants, the expression of both genes was increased in response to the ABA treatments, whereas it was decreased in the ABA-biosynthesis mutant *aba1-5*. Using protoplast transfection assays, the authors also determined that both *SVB* and *SVB2* had similar expression patterns, as well as a similar protein subcellular localisation. In addition, they analysed the trichome numbers in the *svb* and *svb2* single and double mutants and in the *SVB* and *SVB2* overexpression plants. The authors conclude that both *SVB* and *SVB2* function redundantly in order to regulate trichome formation in Arabidopsis by affecting some of the key regulatory genes.

Serba et al. [20] describe the molecular resistance mechanisms to a major grain *Sorghum bicolor* (sorghum) pest, the sugarcane aphid *Melanaphis sacchari* (SCA). Using comparative transcriptome analysis, they characterised the differentially expressed genes between the SCA-resistant sorghum genotype TAM428 and its susceptible counterpart Tx2737. They also determined temporal changes in the gene expression during an SCA infestation. Finally, the authors identified the transcription factors that regulate the differentially expressed genes in response to an SCA feeding. Their findings can help to obtain an improved germplasm resistance to SCA.

The article by Pinski et al. [21] is a study of the impact of 3,4-dehydro-L-proline (3,4-DHP) on PCD in *B. distachyon* roots. 3,4-DHP acts as a substrate for various enzymes, including prolyl-4-hydroxylases. Thus, it can be used as a selective inhibitor of the hydroxyproline-rich glycoprotein (HRGP) synthesis, the cell wall EXTs in particular, thus enabling their functions to be elucidated. By using morphological and cytological ultrastructural observations combined with the immunocytochemical detection of selected EXT epitopes, the authors demonstrated that 3,4-DHP induces the features that are typical for the vacuolar-type cell death. They confirmed their study using the TUNEL test, which revealed that there were more nuclei with fragmented DNA in the 3,4-DHP-treated roots than in the control. In addition, the treated roots exhibited increased expressions of two genes encoding metacaspases, which are proteases that play an essential role in PCD in plants. This study highlights the importance of HRGPs in root growth and root hair development.

In their article, Xu et al. [22] describe a transcriptomic profiling analysis that had been performed on the leaf material of the *Oryza sativa* (rice) *purple leaf* (pl) mutant at the grain-filling stage, during which the mutant plants attain a purple colour, and the wild-type plants are characterised by a typical leaf colouration. Using RNA sequencing, the authors identified 609 differentially expressed genes (DEGs) that are linked with regulating the purple colour, of which 513 were upregulated while the remaining were downregulated. They also confirmed their association with phenylpropanoid and flavonoid synthesis and phenylalanine metabolism. These results provide a better understanding of rice leaf colouration and may help manipulate this trait in this cereal crop.

Wang et al. [23] deliver the description of *tt1Δ15aa*, which is a TRANSPARENT TESTA GLABRA1 (TTG1) mutant of Arabidopsis. This WD40-domain-containing protein regulates various aspects of the cell fate that are linked with root and seed development and secondary metabolism. The authors characterised the C-terminus of this protein in the interaction of TTG1 with the basic helix-loop-helix (bHLH) transcription factor GLABRA3 (GL3) and revealed that *tt1Δ15aa* is a *TTG1* loss-of-function mutant. Using transfected protoplasts, they determined that the deletion of the last three C-terminal amino acids, or even the 339L alone, is enough to completely abolish the interaction between TTG1 and GL3. This demonstrates that the C-terminal domain of TTG1 is critical for maintaining its function in Arabidopsis.

In another article, Larriba et al. [24] study the role of metabolism in wound-induced de novo organ formation in hypocotyl explants of *Solanum lycopersicum* (tomato). The authors used detailed transcriptomic and targeted metabolomics analyses to reveal that wounding induced considerable metabolomic reprogramming. The changes they observed were linked with the reactivation of photosynthesis, induced photorespiration and increased glycolysis in some cells in the apical region of the hypocotyls and also enabled rapid proliferation during the initial callus growth. This study provides an original insight into the molecular mechanisms of the wound-induced organ formation of an important crop.

Gajek et al. [25] focus on root hair development in *Hordeum vulgare* (barley) in the penultimate article. While this process is well-studied in Arabidopsis, little is known about root hair morphogenesis in monocots. Based on their TILLING barley population that had been developed using chemical mutagenesis, the authors identified and characterised the *root hair primordium 1.e* (*rhp1.e*) mutant with extremely short root hairs. Using whole-exome sequencing, they identified a candidate gene, *H. vulgare CELLULOSE SYNTHASE-LIKE C1* (*HvCSLC1*), which is responsible for the mutant phenotype. They confirmed the role of this gene in root hair development and predicted that it encodes glucan synthase, which can be involved in xyloglucan biosynthesis in barley. Thus, this study extends the understanding of root hair elongation in barley.

The final publication of this Special Issue gives an insight into how Ning et al. [26] investigate the role of the cell wall homogalacturonans (HGs) in the development and ripening of the *Musa* AAA (Dwarf Cavendish banana) fruit. Using transcriptomic approaches, the authors found that most of the HG-modifying genes in banana peel were underexpressed during fruit development. Almost all of the HG-modifying gene families had differential expression patterns after ethylene treatment. They also revealed that most of the top genes in the Core Enrichment Genet Set are involved in the degradation of HG. After fruit softening, all of them were upregulated, which paralleled an increase in the activities of the degradation of the HG enzymes. One of the exciting findings was the occurrence of active HG biosynthesis during the fruit ripening process. The authors conclude that the HG-modifying genes are involved in the expansion and cell growth in the banana peel during fruit development and play a pivotal role in the disassembly of pectin polysaccharides in fruit ripening.

The earlier interest of the research community in Special Issues on “Plant Cell and Organism Development” has encouraged us to continue as the Topic Collection on “Advances in Plant Cell and Organism Development”: https://www.mdpi.com/journal/ijms/special_issues/Advances_Plant_Development (accessed on 1 February 2022). We hope that it will be as successful as both editions of the Special Issue.

## Data Availability

Not applicable.
